# Fractal analysis of collision cascades in pulsed-ion-beam-irradiated solids

**DOI:** 10.1038/s41598-017-17781-5

**Published:** 2017-12-14

**Authors:** J. B. Wallace, L. B. Bayu Aji, L. Shao, S. O. Kucheyev

**Affiliations:** 1 Lawrence Livermore National Laboratory, Livermore, 94550 California USA; 2 Department of Nuclear Engineering, Texas A&M University, College Station, 77843 Texas USA

## Abstract

The buildup of radiation damage in ion-irradiated crystals often depends on the spatial distribution of atomic displacements within collision cascades. Although collision cascades have previously been described as fractals, the correlation of their fractal parameters with experimental observations of radiation damage buildup remains elusive. Here, we use a pulsed-ion-beam method to study defect interaction dynamics in 3*C*-SiC irradiated at 100 °C with ions of different masses. These data, together with results of previous studies of SiC and Si, are analyzed with a model of radiation damage formation which accounts for the fractal nature of collision cascades. Our emphasis is on the extraction of the effective defect diffusion length from pulsed beam measurements. Results show that, for both Si and SiC, collision cascades are mass fractals with fractal dimensions in the range of ~1–2, depending on ion mass, energy, and the depth from the sample surface. Within our fractal model, the effective defect diffusion length is ~10 nm for SiC and ~20 nm for Si, and it decreases with increasing cascade density. These results demonstrate a general method by which the fractal nature of collision cascades can be used to explain experimental observations and predict material’s response to radiation.

## Introduction

Bombardment of crystals with energetic ions inevitably creates lattice defects. An energetic particle propagating through a crystalline material transfers a part of its kinetic energy through elastic collisions with nuclei of the target, creating atomic displacements. The target atoms displaced by the bombarding particle can have sufficient energy to collide with other lattice atoms and create subsequent generations of atomic displacements. The cumulative result of such successive generations of collisional processes is the formation of a ballistic cascade of point defects: vacancies and interstitials as well as a relatively small number of antisite defects in compound targets^1^. With increasing ion or target mass, the cross section of elastic scattering increases, leading to larger volumetric densities of atomic displacements within cascades^1–3^. As a result, for a given target material and ion energy, light ions tend to create dilute collision cascades, while heavier ions create denser cascades^1–3^.

For most radiation conditions relevant to the performance of nuclear materials and to ion-beam-processing of semiconductor devices, the mobile point defects ballistically generated in collision cascades can interact with each other and with the other lattice imperfections through so called dynamic annealing (DA) processes, involving point defect recombination and clustering^3^. Numerous previous studies have shown that, even for cases when defect production by electronic excitation is negligible, the damage production efficiency and DA can depend strongly on the mass and energy of bombarding particles and, hence, on the geometry of collision cascades^1,2^. Such influence of the three-dimensional (3D) shape of collision cascades on radiation damage processes is one of the most complex aspects of radiation defect physics. Despite decades of research, our understanding of cascade density effects on DA remains limited even for Si, which is arguably the simplest and most extensively studied material^4^.

Limitations in the understanding of cascade density effects arise to some extent from the complex 3D geometry of collision cascades. Indeed, even the definition of the cascade density is not straightforward and has been debated over the past several decades^2,5–9^. In the 1980s, great progress toward understanding geometry of collision cascades was made by Winterbon *et al*.^10,11^, Cheng *et al*.^12^, and Rossi *et al*.^13^. They^10–13^ showed that the distribution of displacements within a collision cascade could be described within the framework of fractal geometry^14^. A number of subsequent theoretical studies were focused on computations of fractal dimensions of (sub)cascades at different stages of their evolution and on understanding limitations of the fractal approach^15–22^.

Despite the accumulated evidence^8,10–13,15–22^ that, in many practical cases, collision cascades are, in fact, fractals, the correlation of the fractal parameters of cascades with experimental observations of a pronounced effect of ion mass and energy on the DA efficiency has remained elusive. In this paper, we attempt to fill in this gap by presenting a fractal-based description of damage formation during irradiation with pulsed ion beams. We find that, at least for Si and SiC bombarded with 500 keV ions with masses ranging from ^4^He to ^238^U, collision cascades are indeed mass fractals over a relatively large length scale range of ~0.5–30 nm, with fractal dimensions of ~1–2. We further demonstrate that our fractal model can be used to analyze results of pulsed ion beam measurements^4,23,24^ for determining an effective diffusion length (${L}_{d}^{fractal}$) of the mobile defects that dominate DA processes. We present new ion-mass-dependent pulsed-beam data for 3*C*-SiC, which is a material of importance to electronic and nuclear applications^25,26^. In addition, we use the fractal model to analyze data from previous pulsed-beam studies of Si and 3*C*-SiC^4,24^. Our results reveal that, for both Si and 3*C*-SiC, ${L}_{d}^{fractal}$ slightly decreases with increasing cascade density.

## Results and Discussion

### Fractal analysis of collision cascades

#### Calculation of ballistic displacements

As in our recent work^27^, we used the Monte-Carlo TRIM code (version SRIM-2013.00, full cascade calculations)^28^ to compute depth profiles and 3D distributions of ballistically-generated lattice vacancies in Si and SiC. In these calculations^27^, we assumed atomic concentrations of SiC and Si of 9.64 × 10^22^ cm^−3^ and 5.0 × 10^22^ cm^−3^, respectively. The threshold energies for atomic displacement (*E*_*d*_) were 15 eV for Si and 20 and 35 eV for C and Si sublattices of SiC, respectively. Such TRIM-code simulations^28^ ignore lattice anisotropy and use isotropic *E*_*d*_ values. This is an approximation since *E*_*d*_ values (as well as a number of parameters describing DA) are, in fact, anisotropic^29^. For the irradiation conditions of the present study, collision cascades are dominated by displacements formed by recoils rather than by incoming ions. In this case, the approximation of isotropic (averaged) *E*_*d*_ values appears to be justified since cascades are 3D assemblies of lattice displacements with recoil-generated displacements formed in different crystallographic directions. In future work, it will be interesting to perform a cascade density analysis with methods that can consider *E*_*d*_ anisotropy and effects related to the forward momentum transfer by energetic ions, similar to previous molecular dynamics simulations of Gao and Weber^30^.

**Figure 1:**
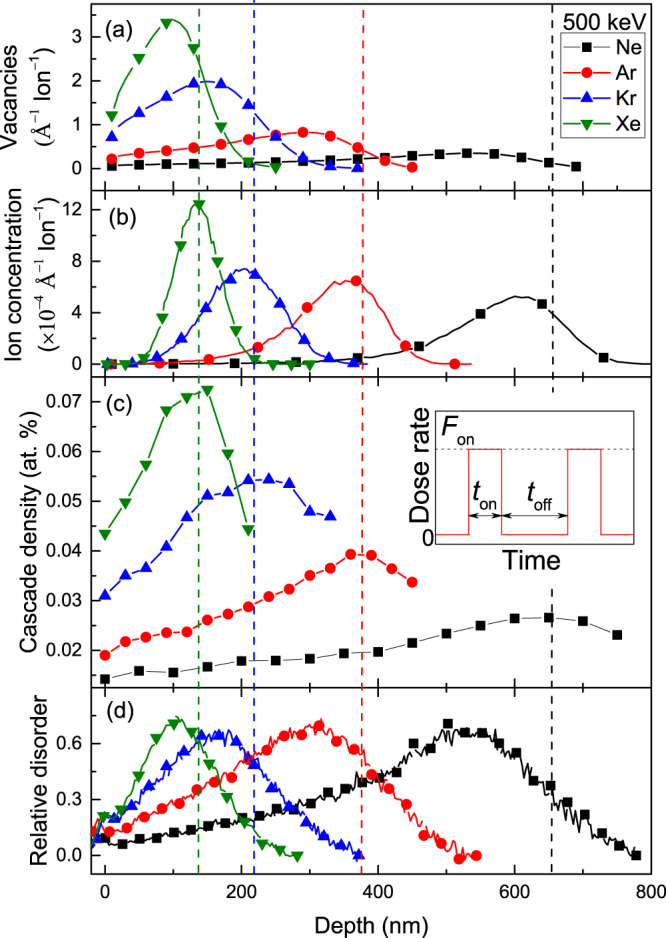
Depth profiles of concentrations of (**a**) ballistically-generated vacancies, (**b**) implanted atoms, and (**c**) collision cascade densities (calculated for *R*_*c*_ = 10 nm) for 500 keV Ne, Ar, Kr, and Xe ion irradiation of SiC. (**d**) Selected depth profiles of relative disorder (measured by ion channeling) in SiC bombarded at 100 °C with continuous beams (i.e., *t*_*off*_ = 0) of 500 keV Ne, Ar, Kr, and Xe ions with a dose rate of 1.9 × 10^13^ cm^−2^ s^−1^ to doses of 11×, 3.5×, 1.7×, and 0.94 × 10^14^ cm^−2^, respectively. The inset in (c) shows the time dependence of the instantaneous dose rate for pulsed beam irradiation, defining *F*_*on*_, *t*_*on*_, and *t*_*off*_. The legend in (a), correlating the symbol type and ion species, applies to all four panels.

Figure 1(a) and (b) show calculated depth profiles of lattice vacancies and implanted atoms in SiC bombarded with 500 keV Ne, Ar, Kr, and Xe ions. All four vacancy profiles in Fig. 1(a) exhibit expected unimodal Guassian-like shapes. We will refer to the positions of vacancy profile maxima as *R*_*pd*_s. The maxima of ion distributions in Fig. 1(b) are located at larger depths than the respective *R*_*pd*_s in Fig. 1(a). All these observations are expected for ion ballistics^4,27,28^.

#### Calculation of fractal parameters of cascades

**Figure 2:**
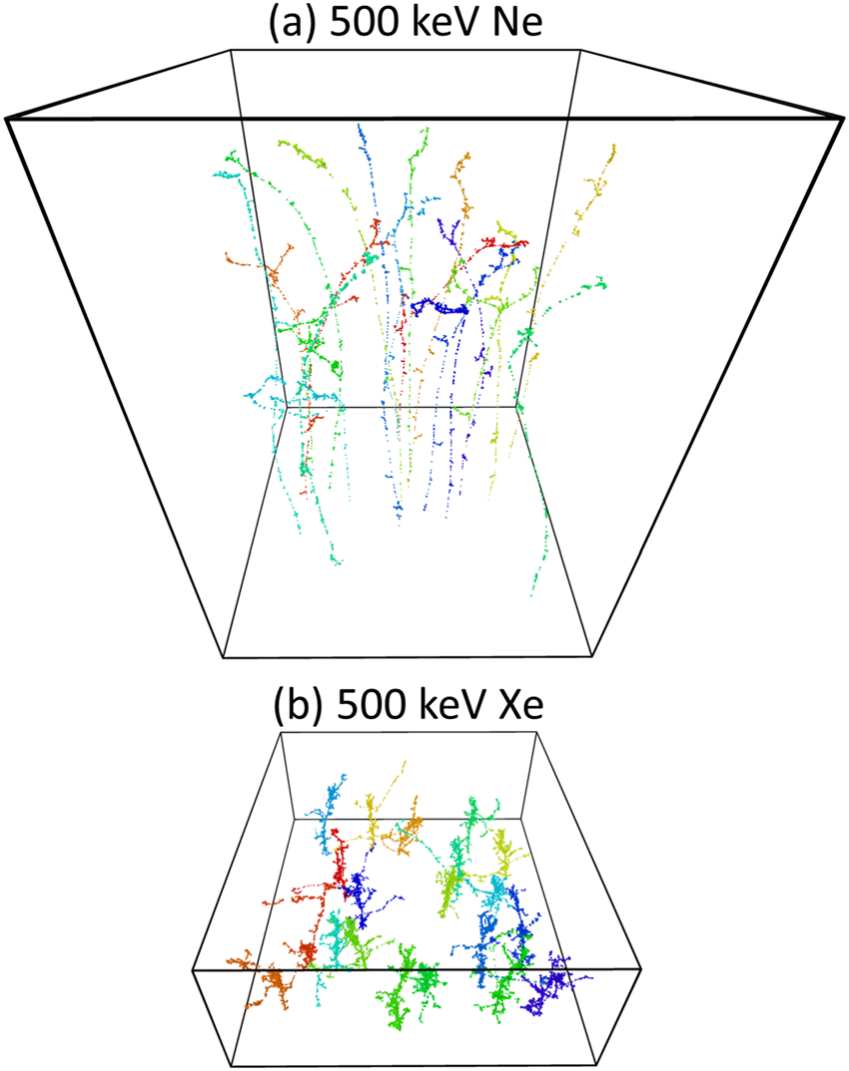
Distribution of lattice vacancies ballistically generated in SiC by bombardment with 500 keV Ne or Xe ions to a dose of 8 × 10^11^ cm^−2^. In both (**a**) and (**b**), the width of the simulation cell is 500 nm. Ion impact points are at the bottom of each simulation cell.

Figure 2 illustrates the shape of ballistic collision cascades (calculated with the TRIM code)^28^ created in SiC by bombardment with light [Ne, Fig. 2(a)] and heavy [Xe, Fig. 2(b)] ions to a dose of 8 × 10^11^ cm^−2^, with ion impacts randomly distributed on the sample surface (at the bottom faces of simulation cells). Figure 2 clearly illustrates the above statement that displacement cascades are dilute and dense for light and heavy ions, respectively. It is also seen that, for a small dose (Φ), corresponding to an average lateral distance between ion impacts of $\frac{1}{\sqrt[]{{\rm{\Phi }}}}\sim 112$ nm, individual cascades are mostly isolated with limited overlap of cascade branches.

**Figure 3:**
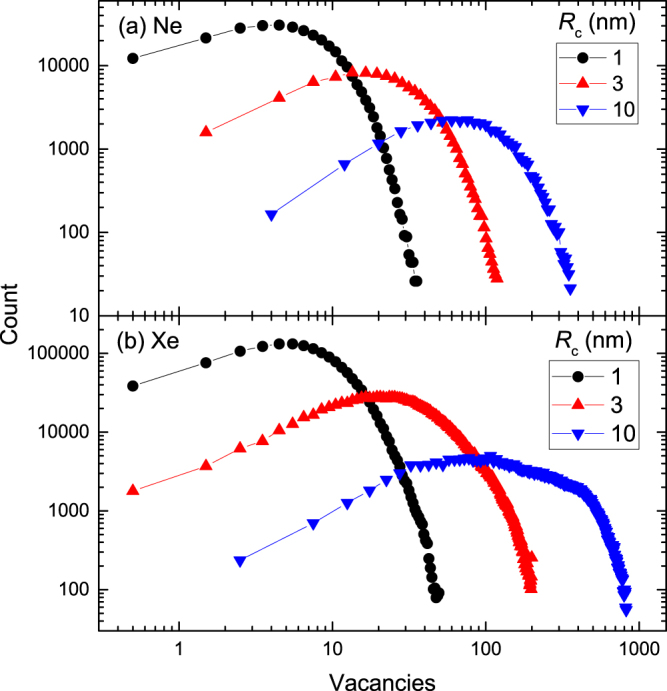
Histograms for vacancies that have a certain number of neighboring vacancies within a sphere of radius *R*_*c*_ for 500 keV (**a**) Ne and (**b**) Xe ion bombardment of SiC at depths corresponding to positions of the maxima of nuclear energy loss profiles (*R*_*pd*_s) and for three different values of *R*_*c*_, given in the legends.

In order to calculate fractal parameters and volumetric densities of vacancies in collision cascades (*ρ*), we analyzed 3D distributions of ballistically-generated vacancies with an algorithm similar to that used by Heinisch and Singh^5^. First, for every vacancy in a cascade, we calculated^31^ the number of neighboring vacancies (*N*_*v*_) whose coordinates lie within a sphere of radius *R*_*c*_ centered on that vacancy. After that, the target was divided into slices of equal widths ($\mathop{ < }\limits_{ \tilde {}}10$% of the *R*_*pd*_) along the incident beam direction, and a database was created for *N*_*v*_ values for vacancies in every depth slice for multiple (typically ≳600) individual cascades. Figure 3 illustrates histograms of the number of neighboring vacancies for slices centered on* R*_*pd*_s for SiC bombarded with 500 keV Ne [Fig. 3(a)] or Xe [Fig. 3(b)] ions. It is seen from Fig. 3 that the histograms are bell-shaped. They broaden and their maxima shift to larger *N*_*v*_ values with increasing either *R*_*c*_ or ion mass.

**Figure 4:**
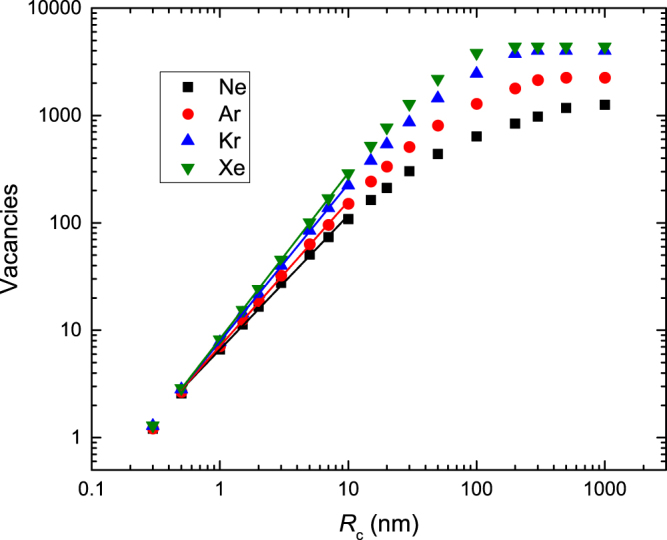
Average number of neighboring vacancies (〈*N*_*v*_〉) within a sphere with a radius of *R*_*c*_ as a function of *R*_*c*_ for 500 keV Ne, Ar, Kr, and Xe ion bombardment of SiC for depths of the maxima of nuclear energy loss profiles (*R*_*pd*_s). Solid lines are linear fits used to determine the fractal dimension and the structure factor.

From such histograms (Fig. 3), we calculated average *N*_*v*_ values (〈*N*_*v*_〉) for every depth slice as a function of *R*_*c*_. Figure 4 shows examples of 〈*N*_*v*_〉 (*R*_*c*_) dependencies for cascades created in SiC by 500 keV Ne, Ar, Kr, and Xe ions at their corresponding *R*_*pd*_s. It is seen that, when depicted on a double-logarithmic plot, 〈*N*_*v*_〉 (*R*_*c*_) dependencies are linear for all the ion masses studied in a relatively wide range of *R*_*c*_ values of ~0.5–30 nm. This is the behavior of a mass fractal when^14^

1 $$\langle {N}_{v}\rangle ={k}_{\circ }{(\frac{{R}_{c}}{{R}_{norm}})}^{D},$$

where *D* is the fractal dimension, *R*_*norm*_ is a normalization radius, and *k*_°_ is the structure factor that represents 〈*N*_*v*_〉 for *R*_*c*_ = *R*_*norm*_. Straight lines in Fig. 4 are linear fits with Eq. 1. Importantly, 〈*N*_*v*_〉 (*R*_*c*_) curves of Fig. 4 do not reveal any evidence of multifractality (for given ion mass and depth from the surface), significantly simplifying the mathematical treatment of cascade geometry^11–13,16–21^.

**Figure 5:**
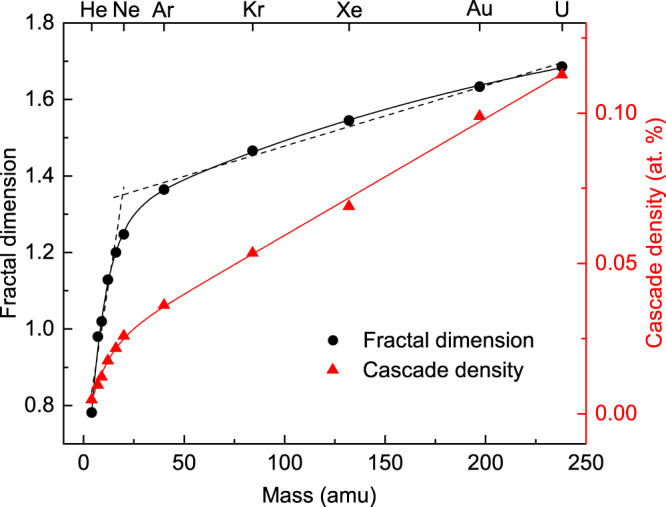
Ion mass dependence of (left axis) the fractal dimension and (right axis) the cascade density (for *R*_*c*_ = 10 nm) for cascades created by 500 keV ions in SiC at their respective *R*_*pd*_s. Dashed lines are to guide the eye.

The ion mass dependence of *D* is shown in Fig. 5 (left axis) for ion masses from ^4^He to ^238^U. It reveals that, for all the ion masses considered, cascades are indeed mass fractals (i.e., *D* < 3), with *D* in the range of ~0.8–1.7 at *R*_*pd*_s. It is also seen that *D* monotonically increases with increasing ion mass and exhibits two well-defined close-to-linear regimes for masses above and below that of Ne.

Ballistic processes in the two sublattices of SiC are different^28,29^. Figure 5 shows results of the fractal analysis of the total vacancies produced, reflecting average behavior of vacancies in both Si and C sublattices. To clarify the contribution from the two sublattices of SiC, we have performed sublattice-resolved calculations of *D* for vacancies produced by 500 keV Ar ions. Such calculations have revealed similar *D* values for all three cases of the total Si + C vacancies (*D* = 1.35), the C sublattice vacancies (*D* = 1.39), and the Si sublattice vacancies (*D* = 1.29).

We define the collision cascade density *ρ* as the average local density of lattice vacancies within the sphere with a radius of *R*_*c*_ at a given depth. Figure 5 (right axis) shows the mass dependence of *ρ* for *R*_*c*_ = 10 nm. It is seen from Fig. 5 (right axis) that *ρ* monotonically increases with ion mass and follows a similar dependence to that of *D* with two close-to-linear regimes with very different slopes, intersecting at a mass close to that of Ne.

In addition to ion mass, *ρ* depends on ion energy and the depth from the sample surface. Figure 1(c) illustrates depth dependencies of *ρ* for bombardment of SiC with 500 keV Ne, Ar, Kr, or Xe ions. It is seen that, for all four ion masses shown, *ρ* at the *R*_*pd*_ is almost twice as large as that near the sample surface. Hence, when different depths are considered, bombardment with different mass ions could be characterized by the same *ρ*. The depth dependence of *ρ* is pronounced and should be taken into account when depth-resolved experimental data is interpreted.

Another important result illustrated by Fig. 1 is that the peak of the depth profile of *ρ* occurs deeper in the material than the maxima of either the vacancy or ion concentration profile. This finding could explain the puzzling observations of numerous previous studies revealing a shift in the position of the bulk defect peak for irradiation conditions with pronounced DA in several materials, including 3*C*-SiC^32^, 4*H*-SiC^33^, GaN^34^, ZnO^35^, MgO^36^, ZrC^37^, TiC^37^, and UO _2_^38^. The fact that such a bulk defect peak shift is negligible for samples irradiated at low temperatures, when DA is suppressed, indicates that this shift is not an ion channeling measurement artifact caused by a dependence of the stopping power of analyzing He ions on the level of lattice disorder^39^, at least for cases of 3*C*-SiC and GaN for which this effect has been extensively studied^32,34^. Depth profiles of ballistically generated displacements, *ρ*, and stable lattice disorder (measured experimentally), such as shown in Fig. 1(a),(c) and (d), respectively, are related. In addition to defect diffusion effects, for any given depth slice, the concentration of stable lattice defects (measured experimentally) depends (i) on the local concentration of ballistic displacements produced and (ii) on the *ρ*-related (depth-dependent) damage formation efficiency. The first contribution (from the concentration of ballistic displacements) reflects the nonlinearity of the damage accumulation behavior. Depth regions with different concentrations of ballistic displacements have received effectively different doses in units of displacements per atom (DPAs) and, hence, are expected to have different damage buildup efficiencies. More efficient damage accumulation is expected to occur in the depth region around the *R*_*pd*_ that is characterized by a larger effective dose (in DPAs). Regimes with larger DA are often characterized by an increase in the nonlinearity of damage buildup curves that become highly sigmoidal, reflecting very rapid damage buildup after a certain dose or damage level is reached. This influence of damage buildup nonlinearity could, for example, explain observations that damage–depth profiles become narrower with increasing DA efficiency at higher sample temperatures^32^. It, however, cannot explain a shift of the damage peak to larger depths.

The bulk defect peak shift can be explained by the second contribution from the *ρ*-related damage formation efficiency that reflects the fraction of ballistic displacements that contribute to the creation of stable lattice damage rather than annihilate via DA processes. Regions characterized by larger *ρ* values (at depths larger than the *R*_*pd*_; see Fig. 1) will exhibit more efficient damage buildup in irradiation regimes characterized by strong DA (which also includes 3*C*-SiC but at temperatures $\mathop{ > }\limits_{ \tilde {}}200$ °C, which is larger than the temperature studied in the present work)^32^. As a result, for regimes with pronounced DA, the peak of the damage–depth profiles measured experimentally could shift to larger depths compared to the peak of the nuclear energy loss profile.

### Correlation of fractal parameters of cascades with experimental data: Estimation of defect diffusion lengths

Figure 1(d) shows representative experimental depth profiles of relative disorder in SiC for 500 keV Ne, Ar, Kr, or Xe ion bombardment at 100 °C. For all these cases, damage peaks are bimodal, with the first small peak at the sample surface and the second major peak in the crystal bulk. Positions of bulk peaks in Fig. 1(d) are at the corresponding *R*_*pd*_s [see Fig. 1(a)]. These observations are consistent with results of several previous studies^27,32,40^.

In order to link the above fractal description of collision cascades with experimental data such as shown in Fig. 1(d), we correlate the dependence of the (measured) damage level in the bulk (*n*) with (calculated) cascade densities (*ρ*). Previous studies for Si^2,4,9,41,42^, Ge^2^, GaN^8,43^, and SiC^27^,^44^ have shown that the damage formation efficiency increases with increasing *ρ*.

**Figure 6:**
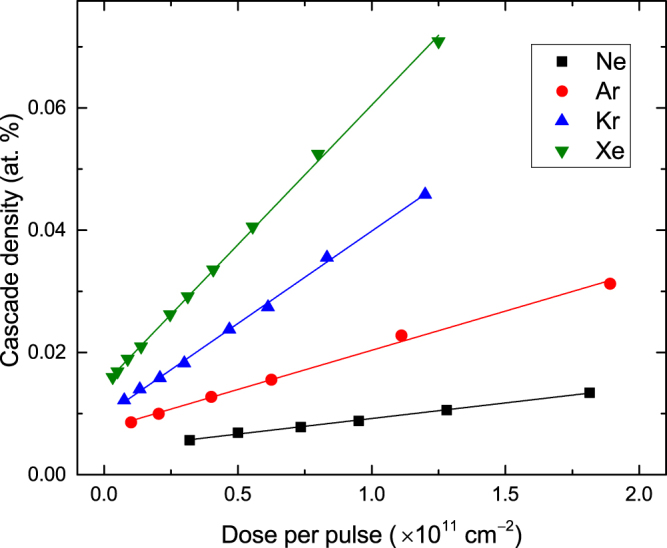
Dose dependencies of the cumulative density of “super-cascades” created in SiC by pulses of 500 keV Ne, Ar, Kr, or Xe ions. Results are for *R*_*c*_ = 30 nm and at depths of *R*_*pd*_s. Solid lines are linear fits.

To evaluate *n*(*ρ*) dependencies, we consider a series of pulsed ion beam experiments with ${t}_{off}\gg \tau$ and all the irradiation parameters constant, except for *t*_*on*_. The inset in Fig. 1(c) is a schematic of the time dependence of the instantaneous dose rate for pulsed beam irradiation. For these pulsed-beam irradiation conditions, the interaction of mobile defects generated in different pulses is suppressed (since ${t}_{off}\gg \tau$), and the *n*(*t*_*on*_) dependence arises from inter-cascade DA processes within the same pulse. We further notice that, for *t*_*on*_ < *τ*, the network of superimposed cascades produced by every pulse (with a dose per pulse of Φ_*pulse*_ = *F*_*on*_ *t*_*on*_) could be considered as a “*super-cascade*,” as illustrated in Fig. 2. For such super-cascades, *ρ* increases with Φ_*pulse*_ as *ρ* = *ρ* ^°^(*R*_*c*_) + *gϕ*_*pulse*_, where *ρ* ^°^(*R*_*c*_) is the density of an isolated ion cascade and *g* (in units of (vacancies)/(ion cm)) is the generation rate of vacancies [such as shown in Fig. 1(a)]. Figure 6 shows an example of calculated *ρ*(Φ_*pulse*_) dependencies for 500 keV Ne, Ar, Kr, and Xe ions, illustrating their linearity.

To simplify the analysis, we consider only the range of small changes to *ρ* when the *n*(*ρ*) dependence is linear. This is the regime of pulsed-beam irradiation with low Φ_*pulse*_ values, when *n* scales with *ρ* as

2 $$n=\sigma {\rm{\Phi }}\rho =\sigma {\rm{\Phi }}(\frac{3{k}_{\circ }{R}_{c}^{D-3}}{4\pi {R}_{norm}^{D}}+g{{\rm{\Phi }}}_{pulse}),$$

where *σ* is the effective cross-section of stable defect formation.

Although dense collision cascades could involve collective energy spike phenomena (thermal and displacement spikes)^2^, we focus here on the *ρ* dependence of DA. The effect of *ρ* on the DA efficiency is related to point defect clustering and annihilation processes that are non-linear; i.e., whose rates depend on concentrations of mobile defects. A concentration is the number of defects averaged over a certain volume. Due to the fractal nature of cascades (i.e., *D* < 3), *ρ* depends on the choice of the averaging radius *R*_*c*_. Moreover, *R*_*c*_ determines the relative difference between *ρ* for different ion masses since *D* is ion mass dependent (Fig. 5). Hence, in contrast to optimistic statements of Heinisch and Singh^5^, this definition of *ρ* (see the previous section) does not avoid the difficult task of the description of cascade volumes. What is the length scale over which defect distributions should be averaged to obtain concentrations of relevance to DA? Such a relevant length scale is the distance over which the diffusion of the point defects dominating DA occurs. In order to differentiate it from the previously used definitions of *L*_*d*_^4,23,24,45^, we will refer to such a distance as ${L}_{d}^{fractal}$, which is a characteristic length for defect diffusion that determines the maximum spatial range over which the mobile point defects dominating DA processes “communicate” with each other.

**Figure 7:**
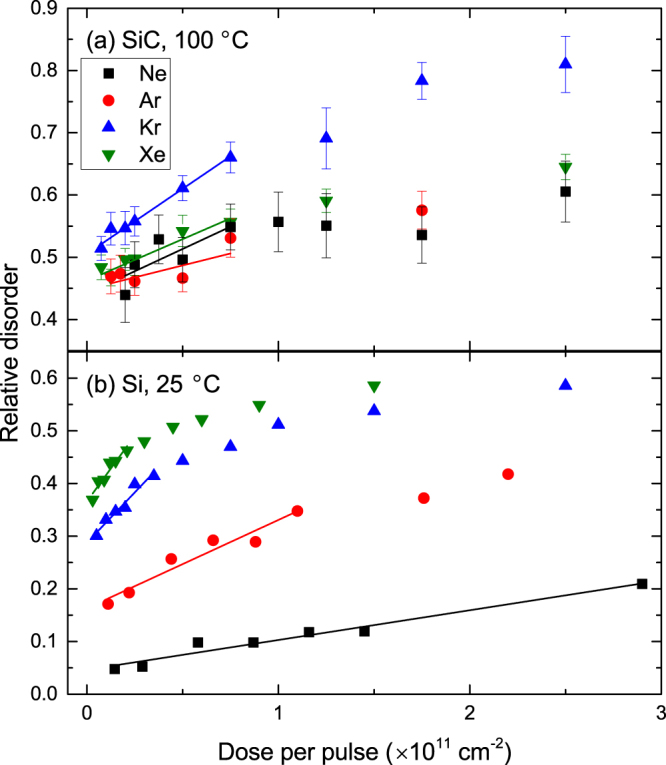
Dose dependencies of relative disorder at the maximum of the bulk defect peak in (**a**) 3*C*-SiC bombarded at 100 °C and (**b**) Si bombarded at 25 °C with pulsed beams of 500 keV Ne, Ar, Kr, and Xe ions, as indicated in the legend in (**a**). For every curve, the dose per pulse was varied by changing *t*_*on*_. Solid lines are results of linear fitting with Eq. 2.

**Figure 8:**
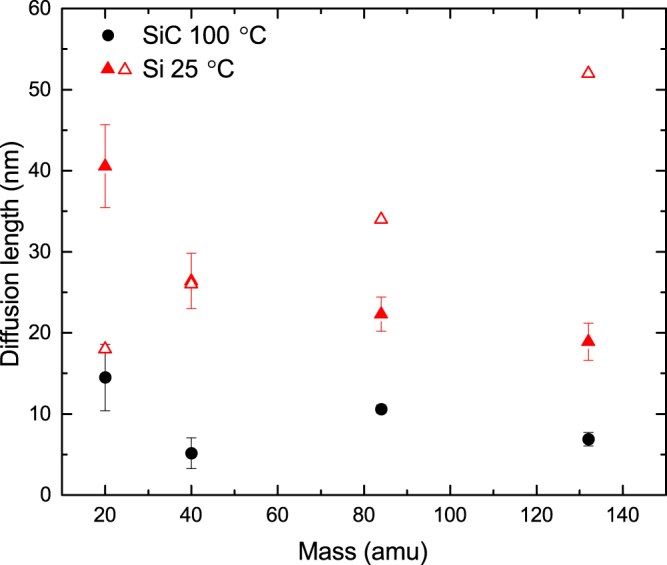
Ion mass dependence of the effective defect diffusion length (${L}_{d}^{fractal}$, solid symbols) for 3*C*-SiC at 100 °C and Si at 25 °C bombarded with 500 keV Ne, Ar, Kr, or Xe ions. Open triangles show previous estimates of *L*_*d*_ for Si from ref.^4^.

Figure 7 shows *n*(Φ_*pulse*_) dependencies for 500 keV Ne, Ar, Kr, or Xe ion bombardment of [Fig. 7(a)] 3*C*-SiC at 100 °C and [Fig. 7(b)] Si at 25 °C. It shows that, as expected, in the low Φ_*pulse*_ region, all the *n*(Φ_*pulse*_) dependencies are linear, followed by a sub-linear regime. Note that the dose ranges where *n*(Φ_*pulse*_) dependencies are linear are comparable for different ion masses if Φ_*pulse*_ is expressed in units of displacements per atom rather than in units of cm^−2^. Solid lines in Fig. 7 are results of linear fitting with Eq. 2, with *D* from Fig. 5 and with the following two fitting parameters: ${R}_{c}={L}_{d}^{fractal}$ and *σ*. The resulting ${L}_{d}^{fractal}$ values for both SiC and Si are shown in Fig. 8. It is seen that, for 3*C*-SiC at 100 °C, ${L}_{d}^{fractal}$ is ~10 nm and, despite relatively large fitting error bars, appears to decrease slightly with increasing ion mass from Ne to Xe. Similarly, Fig. 8 shows that ${L}_{d}^{fractal}$ for Si at 25 °C is ~20 nm and also tends to decrease with increasing ion mass. Such a decrease in ${L}_{d}^{fractal}$ with increasing ion mass could be attributed to an increased density of defect traps in denser cascades. Indeed, more efficient trapping of mobile defects is expected for denser cascades. In addition, denser cascades could exhibit defect types that act as more efficient mobile defect traps than the defects formed in more dilute cascades, leading to reduced ${L}_{d}^{fractal}$ values for irradiation with heavier ions. Further insight into the dominant DA mechanisms could be provided by future atomistic modeling studies benchmarked against our data.

Figure 8 (open triangles) also shows results of our previous estimates^4^ of the mass dependence of *L*_*d*_ in Si for the same data set. These previous *L*_*d*_ estimates^4^ were based on analyzing *n*(*t*_*on*_) data for low *t*_*on*_ values (i.e., low Φ_*pulse*_) when the average lateral distance between neighboring ion impacts in each pulse is larger than *L*_*d*_. A main assumption of these previous *L*_*d*_ estimates is that, for any given depth slice in the plane parallel to the sample surface, each ion creates a damage zone with a circular area with a radius *L*_*d*_ + *R*_*ballistic*_, where *R*_*ballistic*_ is the radius of the ballistic cascade. An assumption of ${L}_{d}\gg {R}_{ballistic}$ was made^4^, and *L*_*d*_ was estimated as the average distance between ion impacts when *n*(*t*_*on*_) ≈ 2*n*(*t*_*on*_ → 0). Figure 8 shows that, although both *L*_*d*_ and ${L}_{d}^{fractal}$ have similar values (the same order of magnitude), *L*_*d*_ increases, while ${L}_{d}^{fractal}$ decreases with increasing ion mass. We attribute this discrepancy to limitations of the previous *L*_*d*_ estimates^4^ that did not take into account the actual cascade geometry, its fractal nature, and the spatial overlap of cascade branches. The difference between *L*_*d*_ and ${L}_{d}^{fractal}$ is particularly pronounced for cases of light (Ne) and heavy (Xe) ions, the regimes when either the cascade geometry or overlap of nearby cascades is expected to become important.

Both previous (*L*_*d*_) and current (${L}_{d}^{fractal}$) estimates of defect diffusion lengths are in general agreement with literature reports of *L*_*d*_ in Si bombarded with ions or electrons at room temperature^41,46–49^ but are much smaller than a *L*_*d*_ of ~300–2000 nm estimated in previous measurements of Si bombarded to ultra-low doses when defect trapping, determining *L*_*d*_, is less likely to occur^50–53^.

**Figure 9:**
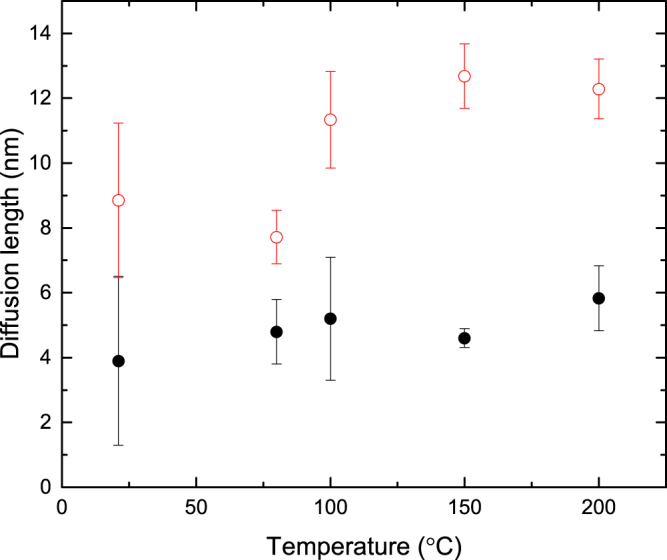
Temperature dependence of the effective defect diffusion length (${L}_{d}^{fractal}$, solid symbols) in 3*C*-SiC bombarded with 500 keV Ar ions. Open circles show previous estimates of *L*_*d*_ from ref.^24^.

Finally, Fig. 9 shows the temperature dependence of ${L}_{d}^{fractal}$ obtained by the fractal analysis of data from ref.^24^ for 3*C*-SiC bombarded with pulsed beams of 500 keV Ar ions. It reveals a ${L}_{d}^{fractal}$ of ~4–6 nm, which increases slightly with increasing temperature in the temperature range studied here (20–200 °C). Open symbols in Fig. 9 show results of previous estimates (with an assumption of cylindrical cascades)^24^ of a *L*_*d*_ of ~10 nm, exhibiting a similarly weak temperature dependence. As for the case of the mass dependence for Si shown in Figs 8 and 9 reveals that ${L}_{d}^{fractal}$ is smaller than the *L*_*d*_ obtained in our previous estimates that did not take into account the actual geometry and overlap of cascades.

## Summary

In summary, we have presented a fractal analysis of collision cascades in ion-irradiated solids. Fractal parameters have been correlated with experimental data on radiation damage in Si and SiC bombarded with pulsed beams of ions with vastly different masses. Results have shown that, for the irradiation conditions studied, collision cascades are mass fractals over a wide range of length scales of ~0.5–30 nm, with fractal dimensions <2. Fractal parameters depend on ion mass, energy, and the depth from the surface. The maximum cascade density occurs at depths larger than those of the maxima of the profiles of nuclear energy loss and implanted ions. This could explain numerous previous observations of a shift of the bulk defect peak to larger depths in irradiation regimes with strong dynamic annealing^32–38^. The effective defect diffusion length (${L}_{d}^{fractal}$) has been evaluated as the relevant length scale over which the fractal collision cascade density is averaged in the analysis of the dependence of the DA efficiency on ion mass. We have found that ${L}_{d}^{fractal}$ is ~10 nm for 3*C*-SiC at 100 °C and ~20 nm for Si at 25 °C. For both materials, ${L}_{d}^{fractal}$ decreases slightly with increasing ion mass (i.e., the cascade density), which can be attributed to an increased density of defect traps in denser cascades. This study links the fractal parameters of collision cascades to experimental data on radiation damage buildup and demonstrates a novel method for measuring effective defect diffusion lengths with the fractal behavior of collision cascades taken into account. This method can be used in future systematic studies of other technologically-relevant materials and irradiation regimes.

## Methods

We used single-crystal epilayers of (001) 3*C*-SiC epitaxially grown on 3-inch-diameter Si substrates at NOVASiC. The epilayers had a thickness of ≳2 *μ*m. The crystal quality of as-received films was verified by measuring a minimum 2 MeV He ion channeling yield of ~1.5%, consistent across the wafer. As-received wafers were cleaved to ~5×40 mm^2^ strips. To improve thermal contact, the strips were attached to a Cu sample holder with conductive silver paste. Temperature was measured by two thermocouples thermally anchored (with silver paste) to the sample holder at the opposite ends of the sample strips. Ion-beam heating effects were negligible for the irradiation conditions used in this experiment. Indeed, the temperature of the sample holder, measured by the two thermocouples, increased by ~0.2 °C for the longest irradiation run with a continuous ion beam (i.e., the case with the maximum heat load).

The 4 MV ion accelerator (National Electrostatics Corporation, model 4UH) at Lawrence Livermore National Laboratory was used for both ion irradiation and ion beam analysis. Samples were bombarded at 100 °C with pulsed beams of 500 keV ^20^Ne, ^40^Ar, ^84^Kr, or ^129^Xe ions at 7° off the [100] direction to minimize channeling. All irradiation experiments were performed in a broad beam mode (rather than in a raster mode)^54^. Dose uniformity over the irradiated area (of $\sim 4\times 5$ mm^2^) was within experimental errors of our ion channeling measurements described below ($\sim 5$%). In each irradiation run, the total ion dose (Φ) was split into a train of equal square pulses. This was done by using a pulse generator with rise and fall times of $\mathop{ < }\limits_{ \tilde {}}25$ ns (Directed Energy, Inc., model PVX-4140) to apply high-voltage pulses to a pair of electrostatic plates. Each pulse had the same dose rate (*F*_*on*_) in the range of (2–8) × 10^13^ cm^−2^ s^−1^ and duration (*t*_*on*_) and was separated from the subsequent pulse by *t*_*off*_. The inset in Fig. 1(c) shows a schematic of the time dependence of the instantaneous dose rate and defines pulsing parameters *t*_*on*_, *t*_*off*_, and *F*_*on*_. In these experiments, we kept *t*_*off*_ fixed to 50 ms, which, as demonstrated previously^40^, was much greater than the defect relaxation time constant (*τ*). With *F*_*on*_, *t*_*off*_, and Φ kept constant, a series of samples was irradiated with different *t*_*on*_ values, varied between 0.3 and 20 ms. The Φ s for different ion masses were chosen such that, for continuous beam irradiation (i.e., *t*_*off*_ = 0), the level of average relative bulk disorder (*n*), measured by ion channeling, was in the range of 0.6−0.9 (with *n *= 1 corresponding to full amorphization). Note that these pulsed beam experiments involve essentially an integration of the damage buildup from a “perfect” as-grown crystal (for zero dose) to the damage level achieved for a given set of irradiation conditions. This final bulk damage level varies from $\sim 0.05$ (for some cases of pulsed beam irradiation) to up to $\sim 0.8$ for the worst damaging case of a continuous beam (see Fig. 7). Doses larger than those used in the present study are required for complete lattice amorphization.

The dependence of stable lattice damage on *t*_*on*_ was studied *ex-situ* at room temperature by ion channeling. Depth profiles of lattice disorder in the Si sublattice were measured with 2 MeV ^4^He^+^ ions incident along the [100] direction and backscattered into a detector at 164° relative to the incident beam direction. The diameter of the analyzing He beam was $\sim 1$ mm. Raw channeling spectra were analyzed with one of the conventional algorithms^55^ for extracting depth profiles of relative disorder. Values of *n* were obtained by averaging depth profiles of relative disorder over 15 channels ($\sim 40$ nm) centered on the bulk damage peak maximum. Error bars of *n* are standard deviations. The relative disorder measured by ion channeling reflects direct backscattering of the probing ion beam propagating in a particular axial channel in the lattice. Hence, it is sensitive to interstitial-based defects and to lattice relaxation around vacancies in the sublattice probed (the Si sublattice in the present work). It is a measurement of the long-range order rather than the short-range order that can be evaluated by other experimental techniques as well as by molecular dynamics simulations^30^.
